# Investigation on the Antibacterial and Anti-T3SS Activity of Traditional Myanmar Medicinal Plants

**DOI:** 10.1155/2018/2812908

**Published:** 2018-10-09

**Authors:** Tianhong Li, Dongdong Zhang, Thaung Naing Oo, Myint Myint San, Aye Mya Mon, Pyae Phyo Hein, Yuehu Wang, Chunhua Lu, Xuefei Yang

**Affiliations:** ^1^Key Laboratory of Chemical Biology (Ministry of Education), School of Pharmaceutical Sciences, Shandong University, Jinan 250012, China; ^2^Key Laboratory of Economic Plants and Biotechnology, Kunming Institute of Botany, Chinese Academy of Sciences, Kunming 650201, China; ^3^Southeast Asia Biodiversity Research Institute, Chinese Academy of Sciences, Yezin, Nay Pyi Taw 05282, Myanmar; ^4^Forest Research Institute, Yezin, Nay Pyi Taw 05282, Myanmar

## Abstract

Myanmar has a rich pool of, but less known, medicinal plants with traditional knowledge. In this study, we aimed to investigate the inhibitory activity of traditional Myanmar medicinal plants against the type III secretion system (T3SS) of* Salmonella enterica* serovar Typhimurium UK-1 *χ*8956 and the intestinal disease-caused by microbes including* S. enterica *serovar Typhimurium UK-1 *χ*8956,* Proteusbacillus vulgaris *CPCC 160013*, Escherichia coli* CICC 10003, and* Staphylococcus aureus *ATCC 25923. The EtOH extracts of 93 samples were used to screen the inhibitory activities against the secretion of T3SS effector proteins SipA/B/C/D of* S. enterica* and the antibacterial activity against* S. enterica*,* P. vulgaris, E. coli,* and* S. aureus.* Out of 71 crude drugs traditionally used, 18 were proofed to be effective either on the growth inhibition of tested bacteria and/or as inhibitors for the T3SS. The EtOH extracts of five plants,* Luvunga scandens* (Roxb.) Buch.-Ham. ex Wight & Arn. (My7),* Myrica nagi* Thunb. (My11),* Terminalia citrina* Roxb. ex Fleming (My21),* Thymus vulgaris* L. (My49), and* Cinnamomum bejolghota *(Buch.-Ham.) Sweet (My104), showed potent inhibitory activities against the secretion of T3SS proteins SipA/B/C/D of* S. enterica *serovar Typhimurium UK-1 *χ* 8956.* Mansonia gagei *J.R.Drumm (My3) and* Mesua ferrea* (Roxb.) L. (My10) showed strong antibacterial activities against* P. vulgaris* and* S. aureus.* This study provided the first scientific evidence of T3SS prohibiting and antibacterial properties for the traditional knowledge in Myanmar of using plants as medicines for treating infections and gastrointestinal disease. Further researches are proposed to discover the active chemical compounds and mechanism of* L. scandens* (Roxb.) Buch.-Ham. ex Wight & Arn,* M. nagi* Thunb.,* T. citrina* Roxb. ex Fleming,* T. vulgaris* L., and* C. bejolghota *(Buch.-Ham.) Sweet as antivirulence drugs and the potential of* M. gagei* J.R.Drumm and* M. ferrea* L. as new broad spectrum plant antibiotics.

## 1. Introduction

Globally, the search for antimicrobials has encountered serious challenge of resistance from pathogenic microorganisms to antibiotics. Conventional antibiotics developed to inhibit the growth of pathogenic microbes are generally no more effective over three years of clinical applications. Though the cost to treat infectious diseases and to find new antibiotics has been largely increased during the past decades, little breakthrough has been made [1]. On the one hand, researches are urgently needed to find more novel antibiotics; on the other hand, alternative therapeutics are widely encouraged with great expectation of solving this problem. Inhibiting or blocking the pathogenic microbial virulence that facilitate the invasive and/or cause the damage of host cells is an good example of emerging direction [1, 2].

Gram-negative bacteria, such as* Salmonella* spp.,* Shigella* spp.,* Yersinia* spp., and* Escherichia coli* (EPEC), are the major cause for gastrointestinal diseases. They have a common virulence factor, i.e., the type III secretion system (T3SS) [3, 4]. T3SS is employed by a number of pathogenic bacterium to inject toxins into host cells [5, 6]. Anti-T3SS is the emerging and novel antivirulence strategy to combat pathogens and has no effects on bacterial growth, which might be less likely to generate bacterial resistance to drugs. Since the first report of salicylidene acylhydrazides as T3SS inhibitors in 2003 [7], several more T3SS inhibitors were discovered. Those include our recent reports of three inhibitors for T3SS of* S. enterica*, namely, fusaric acid, licoflavonol, and Csn-B [8–10]. Despite this progress, systematic screenings on the inhibitors of T3SS either from known chemicals or from natural medicinal plants are desired; and the quest of underlying mechanisms is increasing.

As a major cause of death to global population [11], infectious diseases are much more serious in the tropical regions such as Southeast Asian, South Asian, and Africa where the warm and humid environment favored the growth and propagation of microorganisms. In particular, Myanmar is one of the countries with high risk of infectious microbial diseases [12], where gastrointestinal ones including diarrhea and dysentery, fever, malaria, and tuberculosis are prevailing. Despite general lack of health data in Myanmar, a study has reported the imposing threat from diarrhea to children, causing 21% of child death in Myanmar [13]. Recently an inspection to poultry products, the major food vector for Salmonella infection, in Yangon market revealed an extremely high prevalence of* Salmonella*, with 97.9% of the sample carrying this bacterium [14]. Alarmingly, among the 138 bacterial isolates, many of them showed different degree of resistance ranging from 70.3% of trimethoprim-sulfamethoxazole to 0.7% of norfloxacin [14]. These pieces of information indicated that solutions of combating Salmonella infection are pressingly required in Myanmar.

In contrary to its high risk of gastrointestinal infection to the population, Myanmar is endowed with rich plant resources and traditional knowledge that has been used for generations for treating various ailments. The* Medicinal Plant List of Myanmar*, the first comprehensive book published by FAME Company, the most famous drug company in Myanmar, compiled a list of more than 1500 species used in Myanmar [15]. Traditional Myanmar medicine uses a wide variety of plants in the treatment of gastrointestinal disorders. However, no literature is available on recording these medicinal plants.

Since December of 2015, we implement continuous market surveys on documenting the medicinal plants sold and recording their traditional knowledge at Zay Cho Market in Mandalay, Myanmar. We acquired 93 dried medicinal plant samples belonging to 51 families (Unpublished Data) with good traditional knowledge for evidence-based scientific explorations in the direction of antibacterial, antioxidant, and antidiabetes properties. In this study, we focus on validation of these medicinal plants on antibacteria properties on (1) antibacterial activity against* Salmonella enterica *serovar Typhimurium UK-1 *χ*8956,* Proteusbacillus vulgaris *CPCC 160013*, Escherichia coli* CICC 10003, and* Staphylococcus aureus *ATCC 25923 and (2) the inhibitory activity on the effector proteins SipA/B/C/D of T3SS of* S. enterica *serovar Typhimurium UK-1 *χ*8956. To our knowledge, this is the first report on scientific validation of traditional medicinal plants on treating bacteria in Myanmar. These results serve an important start to select evidence-based Myanmar medicinal plants as drug sources for the development of the plant antibiotics and inhibitors of* Salmonella* T3SS.

## 2. Materials and Methods

### 2.1. Collection and Identification of Medicinal Plant Materials

The 93 samples of medicinal plants were purchased from Zay Cho Market in April 2016 after a preliminary ethnobotanical survey in December 2015. The samples were requested through Myanmar name recorded in the Medicinal Plant List of Myanmar [15], from which Latin names of each sample were also noted. Each sample was double checked with the sellers and noted with its Myanmar name and Latin name. The samples were crosschecked with various ethnobotanists and taxonomists (Professor Shengji Pei, Dr. Jie Cai, Ms. Jun Yang, and Mr. Yu Zhang from Kunming Institute of Botany, Daw Myint Myint San and U Aung Zaw Moe from Forest Research Institute, and Professor Shude Yang from Yunnan University of TMC) based on macroscopical features of the materials and personal experience. Further identification with voucher specimens in the lab was carried out when taxonomic confusions exist. The final adoption of Latin names was checked and used based on the information provided from the Plant List (http://www.theplantlist.org/).

### 2.2. Preparation of Extracts

Each plant material was extracted three times with EtOH. The supernatant of each extract was filtered through Whatman No. 2 filter paper and evaporated under reduced pressure at 60°C to afford corresponding crude extracts, respectively. All the 93 EtOH extracts were dissolved in DMSO at the concentration of 20 mg/mL, respectively.

### 2.3. Bacterial Cultivations

*S. enterica *serovar Typhimurium UK-1*χ* 8956 [16] was grown in Luria-Bertani (LB) broth (1% tryptone, 0.5% yeast extract, 1% NaCl, pH 7.4) or on LB agar plates supplemented with 0.2% L-arabinose at 37°C or 25°C with shaking at 220 rpm.* P. vulgaris* CPCC 160013*, E. coli* CICC 10003, and* S. aureus* ATCC 25923 were grown on LB agar media.

### 2.4. Antibacterial Assay

The antibacterial activities of 93 extracts against* S. enterica* serovar Typhimurium UK-*χ*8956,* S. aureus* ATCC 25923,* P. vulgaris* CPCC 160013, and* E. coli* CICC 10003 were measured with a paper disc diffusion assay [17]. Tested extracts were absorbed onto individual paper disks (6 mm diameter) at 80 *μ*g/disc and placed on the surface of the agar media. Kanamycin was used as positive control. The assay plates were incubated at 37°C for 24 h and examined for the presence of inhibition zone.

### 2.5. Measurement of Bacterial Growth

*S. enterica* serovar Typhimurium UK-1*χ*8956 was grown in LB broth with 0.2% L-arabinose at 37°C/220 rpm in a shaker overnight. Then, 1:10 dilutions of overnight cultures of* S. enterica* were grown in LB (0.2% L-arabinose) for 12 hrs with the addition of extracts at the indicated concentrations. OD_570_ of the culture was measured once every hour using a microplate reader (Bio-Rad 680, USA) until 12 hrs. The samples were repeated 3 times in each experiment.

### 2.6. Isolation and Detection of T3SS Effector Proteins

The potential anti-T3SS activities of 93 Myanmar medicinal plant extracts were screened for their effects on the secretion of the SPI-1 effector proteins of* S. enterica* at the concentration of 80 *μ*g/mL (Fig.  S1). Csn-B was used as the positive control [10]. 1:10 dilutions of overnight cultures of* S. enterica* were grown in LB (0.2% L-arabinose) for 4 hrs in the absence or presence of compounds at indicated concentrations at 37°C /220 rpm. Secreted proteins from the supernatant of 1 mL culture were precipitated with a final concentration of 10% TCA at 4°C and centrifuged at 12000* g* for 15 min and then washed with 250 *μ*L ice-chilled acetone. The procedure was repeated 2 times and the precipitates were allowed to dry for 15 min. The pellets were dissolved with loading buffer to an optical density (OD_600_) that ensure each contains equivalent secreted protein. The protein samples underwent protein denaturation heated for 5 min at 95°C and then was separated by 10% SDS-PAGE and stained with Coomassie blue and subsequently detected by Western blotting.

### 2.7. Western Blotting Analysis

To concretely detect SipC or FliC (flagellar protein),* S. enterica *was cultured and treated as described above. The protein samples were mixed with sample buffer and loaded on to a 10% SDS-PAGE. The gels were blotted onto PVDF membranes. Next, the electrophoresis membranes were washed with 5% w/v BSA (bovine serum albumin) in TBST (Tris-buffered saline mixed with Tween 20) at room temperature for more than 1 h with shaking to blocking specific binding. Then, membranes were incubated in 5% w/v BSA containing the specific antibody (like anti-SipC or anti-FliC) overnight at 4°C. The excess antibody was washed off with TBST (5 min, three times), and membranes were incubated for 1 h in TBST containing the secondary antibody. Then, membranes reacted with the first antibody at room temperature with shaking. Then, membranes were washed three times with TBST again. Finally, ECLA reaction buffer (0.1 M Tris-HCl, pH 8.5, 25 mM luminol, 4 mM p-coumaric acid) and ECLB reaction buffer (0.06% v/v H_2_O_2_ in 0.1 M Tris-HCl, pH 8.5) were mixed. Membranes were incubated in the mixture for 2 min, and proteins were detected by ECL method (Molecular Imager ChemiDoc XRSt; Bio-Rad, Hercules, CA). Relative intensity of protein levels was analyzed using Image Lab Software.

## 3. Results

### 3.1. Ethnobotanical Survey of Myanmar Medicinal Plants

The ethnobotanical inventory of medicinal plants in Myanmar enlisted more than 100 plant materials that are widely traded at Zay Cho Market (Unpublished Data). For the 93 species tested in this study, 71 were noted with traditional uses related to anti-infectious functions such as diarrhea, dysentery, digestion, flatulence, fever, and cough. Taking into consideration the screening of more potential agent for anti-T3SS, we included all the materials for the bioactivity tests in this research. Eighteen (Figure 3) out of 93 traditional Myanmar medicinal plants showed evident antibacterial activities including antivirulence. The ethnobotanical information and the results of the tested activities are detailed in Table 1. We also made a brief evaluation of the status of the research of these species based on the retrieved literature using Web of Science (Table 1). It shows that all these species have been found to possess antibacterial property. However, the intensity and level of research differed from species to species. A brief review of the reported chemical constituents of the 18 traditional medicinal plants was also provided in Table S1.

### 3.2. Antibacterial Activities

**Figure 1:**
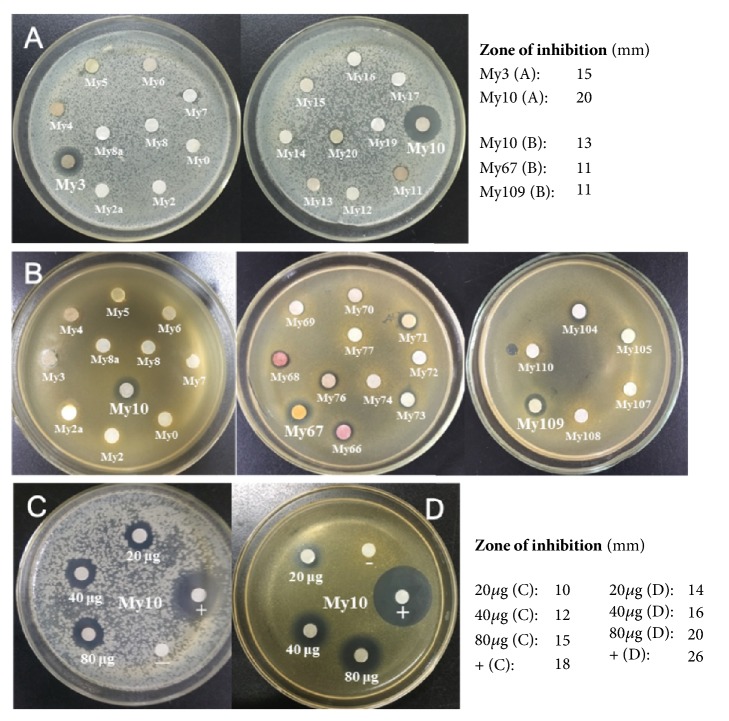
The screening of the antibacterial activity of crude extract of MTMs. (A) My3 and My10 inhibited the growth of* P. vulgaris *CPCC 160013. (B) My10, My67, and MY109 inhibited the growth of* S. aureus *ATCC 25923. (C) The positive dose effects of inhibition on* P. vulgaris* CPCC 160013 for My10 at three concentrations levels (20, 40, 80 *μ*g), with comparison to positive control (+, Ampicillin, 2 *μ*g) and negative control (-, DMSO, 4 *μ*L). (D). The positive dose effects of inhibition on* S. aureus* ATCC 25923 for My10 at three concentrations levels (20, 40, and 80 *μ*g), with comparison to positive control (+, Kanamycin, 10 *μ*g), and negative control (-, DMSO, 4 *μ*L).

The antibacterial activities of 93 extracts were carried out against* S. enterica* serovar Typhimurium UK-1 *χ*8956,* S. aureus* ATCC 25923,* P. vulgaris* CPCC 160013, and* E. coli* CICC 10003, respectively. Results were measured with a paper disc diffusion assay (80 *μ*g/disc).* M. ferrea* L. (My10) significantly inhibited the growth of* P. vulgaris* with inhibitory diameter of 20 mm, followed by* M. gagei* J.R.Drumm (My3), 15 mm (Figure 1(A)). The growth of* S. aureus *was significantly inhibited by* M. ferrea* L. with a diameter of 13 mm and moderately inhibited by* Curcuma comosa* Roxb. (My67, 11 mm) and* Coptis teeta* Wall. (My109, 11 mm) (Figure 1(B)). The inhibitory effects of* M. ferrea *L. on* P. vulgaris* and* S. aureus* are dose-dependent (Figures 1(C) and 1(D)). No tested medicinal plants inhibited the growth of* S. enterica* and* E. coli*.

### 3.3. Inhibition of the Secretion of T3SS Effector Proteins

**Figure 2:**
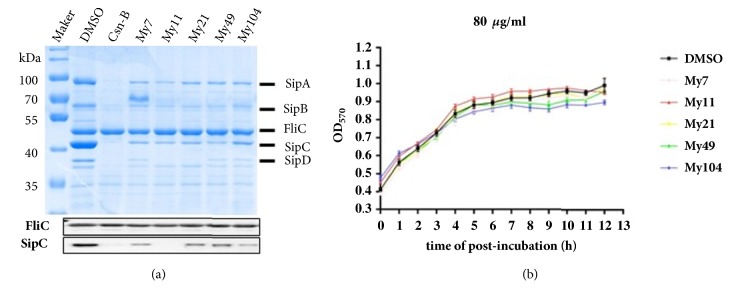
(a) The inhibitory activities of My7, My11, My21, My49, and My104 (80 *μ*g/mL, respectively) against the secretion of the Salmonella pathogenicity island 1 (SPI-1) effector proteins of* S. enterica *serovar Typhimurium UK-1*χ*8956. SipA/B/C/D, SPI-1 effector proteins. (b) The five extracts did not affect the growth of* S. enterica *serovar Typhimurium UK-1 *χ*8956* in vitr*o. DMSO, negative control; Csn-B, positive control (100 *μ*M). FliC, flagellar filament protein; M, marker.

**Figure 3:**
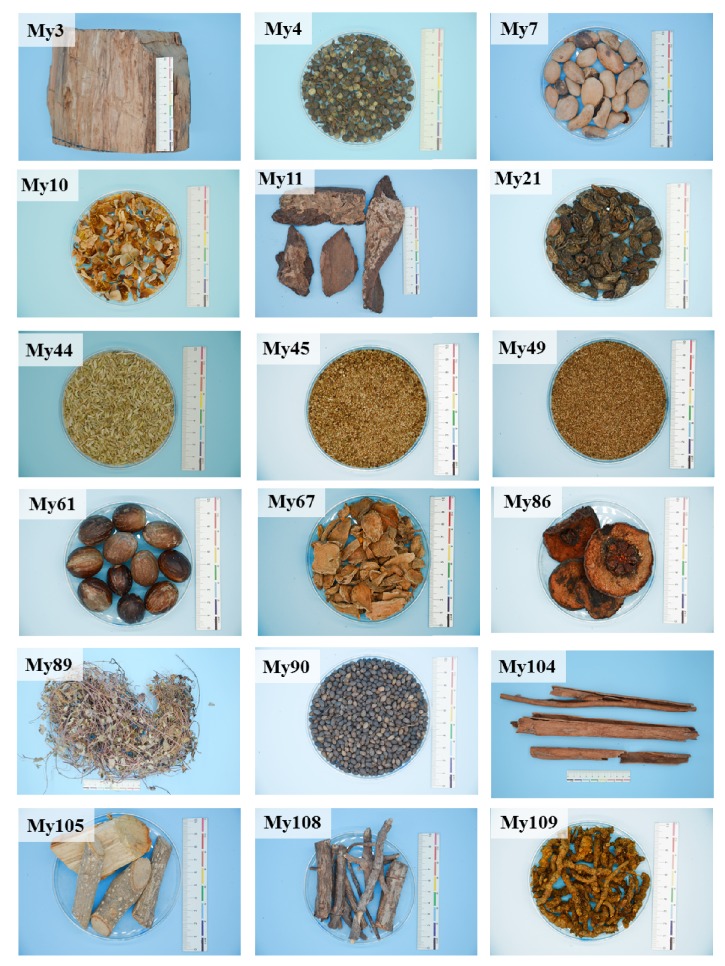
Pictures of 18 crude drugs of traditional medicinal plants with antibacterial and anti-T3SS properties.

Among the 93 tested samples, the extracts of* Luvunga scandens* (Roxb.) Buch.-Ham. ex Wight & Arn (My7),* Myrica nagi* Thunb. (My11),* Terminalia citrina* Roxb. ex Fleming (My21),* Thymus vulgaris* L. (My49), and* Cinnamomum bejolghota* (Buch.-Ham.) Sweet (My104) exhibited strong inhibitory effects on the secretion of the T3SS effectors SipA/B/C/D (Figure 2(a)). None of them had effect on the growth of* Salmonella* (Figure 2(b)). Apart from those,* Litsea cubeba* (Lour.) Pers. (My4),* M. ferrea* L. (My10),* Foeniculum vulgare* Mill. (My44),* Anethum graveolens* L. (My45),* Myristica fragrans* Houtt. (My61),* Garcinia pedunculata* Roxb. ex Buch.-Ham. (My86),* Centella asiatica* (L.) Urb. (My89),* Brucea javanica* (L.) Merr. (My90),* Coscinium fenestratum* (Goetgh.) Colebr. (My105), and* Tylophora indica* (Burm. f.) Merr. (My108) also showed moderate potential inhibitory effects on the secretion of SipA and SipC (Figure S1). Thus,* L. scandens* (Roxb.) Buch.-Ham. ex Wight & Arn (My7),* M. nagi *Thunb (My11),* T. citrina* Roxb. ex Fleming (My21),* T. vulgaris* L. (My49), and* C. bejolghota* (Buch.-Ham.) Sweet (My104) can be selected for further investigation for anti-T3SS active components and the mechanisms of action.

## 4. Discussion

### 4.1. Scientific Evidence of Antibacterial Activity of Traditional Myanmar Medicinal Plants

The results of this study confirmed that the crude extracts of four medicinal plants, i.e.*, M. gagei *J.R.Drumm,* M. ferrea *L.,* C. comosa* Roxb., and* C. teeta *Wall., which were traditionally used in antibacterial purpose (Figure 1, Table 1) in Myanmar, are proofed to be antibacterial with particular inhibitory effects on* P. vulgaris* and* S. aureus*. In addition, evidence of previous reports from neighboring countries consolidated the finding (Table 1).

The crude extract from* M. ferrea* L. (My10) is the most effective one among the previously mentioned four plants on both* P. vulgaris* and* S. aureus*. Traditionally, the dried flowers of* M. ferrea* L. are used for fever, insomnia, palpitation, dizziness, and breathlessness by Myanmar people. This plant is also widely used as a folk medicine for fever, dyspepsia, insomnia, renal and skin care in India [21], and antitumor [35] and anticholinesterase [36] activities in Malaysia. It is reported that 4-alkyl- and 4-phenylcoumarins from the flowers of* M. ferrea* L. were promising agent as multidrug resistant antibacterials, inhibiting a large number of Gram-positive and Gram-negative bacteria [37].

*M. gagei* J.R.Drumm (My3) was reported to be a folk medicine in Thailand used as cardiac stimulant, vertigo, antiemetic, antidepressant, and refreshment agent [38]. Mansonones and coumarins are the main antifungal, antibacterial, antioxidant, antiestrogenic, antitumor, and larvicidal compounds [18, 39–41]. Nevertheless, the taxonomy status of* M. gagei *J.R.Drumm remains unsolved according to the Plant List database, and the research on botany characterization is fundamental for its further scientific investigation.

*C. comosa* Roxb. (My67) are widely used and studied in Thailand. It is used as a food ingredient and for treating gynecological problems [42]. Pharmacological research has shown that this plant has multibioactivities including antilipidemic, choleretic, estrogenic, uterotrophic, anti-inflammatory, male fertility, vascular relaxation, nematocidal, prevention of hepatotoxicity, antioxidant, antiallergic, antibreast and antiuterine effects [43–47]. The effective compounds are mainly sesquiterpenoids and diarylheptanoids [27, 42, 48]. No report has indicated the antibacterial activity of* C. comosa* Roxb. previously. But* C. longa* L. of the same genus were reported to have antibacterial properties [49]. The new bioactivity of* C. comosa* Roxb. found in this research on the inhibitory to* S. aureus* deserves a further research.

As a Myanmar folk medicine,* C. teeta* Wall. (My109) is used along with* Piper nigrum *L. for cough and asthma. In China, it is a popular and well-known medicinal plant widely used for antiulcer, anti-inflammatory, and antibacterial [50]. It contains mainly alkaloids such as berberine BR, coptisine, jatrorrhizine, and worenine and is widely used as antibacterial and antidiarrheal agent for a wide range of bacteria [51, 52]. It is also used for inflammatory eye diseases, decreased vision, cataract, skin-related problems, indigestion, constipation, jaundice, fever especially in malaria, gonorrhea, and urine disorders in India [53].

### 4.2. Screening Potential T3SS Inhibitor from Myanmar Traditional Medicinal Plants

Upon the acknowledgment of the alarming fast biological evolution of resistance to antibiotics, the shift from killing and/or inhibiting pathogenic bacteria to inhibit virulence factors provide a new solution for the treatment of microbial infectious diseases [1]. Anti-T3SS is an effective antivirulence approach, and a number of T3SS inhibitors have been identified in the past decade [2, 54], including salicylidene acylhydrazones,* N*-phenyl-benzamides, thiazolidinones, and phenolic acids. Yet, new T3SS inhibitors are still desired for antivirulence drug development [2]. Myanmar is rich in plant diversity and diverse in the application of traditional medicinal plants, which provides a large resource pool for screening the potential inhibitors of T3SS from traditional medicinal plants. In this study, among the 93 extracts from Myanmar medicinal plants screened, 15 were found as potential T3SS inhibitors, which afforded 5 (5%) with significant potent and 10 (11%) with moderate activity, indicating a high success rate of discovering novel inhibitors of T3SS from traditional Myanmar medicinal plants.

For the 15 medicinal plants with T3SS inhibitory potential, seven of them (*L. cubeba *(Lour.) Pers.,* M. ferrea *L.,* F. vulgare *Mill.,* A. graveolens *L.,* T. vulgaris *L.,* M. fragrans *Houtt., and* C. asiatica *(L.) Urb.) were intensively studied with good number of researches showing their antibacterial properties (Table 1). Although* C. comosa*,* B. javanica*,* C. fenestratum, *and* T. indica* received much attention in the past, relatively less studies were focused on their antibacterial activities (Table 1). In contrast, very few studies were carried out for* L. scandens*,* M. nagi*,* T. citrina*,* G. pedunculata*,* C. bejolghota*, and* C. teeta*, not only in the direction of antibacterial research but also in the genera field of science. Nevertheless, no matter how intensive these species have been studied in the past, none of them were investigated with focus as a T3SS inhibitor. This indicates that a big gap and potential are remained for future investigation of these Myanmar medicinal plants, from which novel approaches and new sources are likely to be discovered in treating drug resistant bacterial.

## 5. Conclusions

In total, 18 out of 93 traditional Myanmar medicinal plants showed evident antibacterial activities including antivirulence, suggesting a great potential of Myanmar medicinal plant resources and the accompanied knowledge systems on combating infectious diseases. The positive results of* M. gagei *J.R.Drumm,* M. ferrea *L.,* C. comosa,* and* C. teeta* against* S. aureus* and/or* P. vulgaris* and* L. cubeba *(Lour.) P*e*rs.* and the effects of M. ferrea *L.,* F. vulgare*,* A. graveolens *L.,* T. vulgaris *L.,* M. fragrans *Houtt., and* C. asiatica *(L.) Urb. as T3SS inhibitors for* S. enterica* serovar Typhimurium UK-1 8956 are worthy of further exploring with priority on identifying their active chemical constituents and to underpin the underlying mechanisms.

## Figures and Tables

**Table 1:** Ethnobotanical information and antibacterial and anti-T3SS activities of the 18 traditional medicinal plants.

**No.**	**Scientific name**	**Family**	**Common name**	**Myanmar name**	**Myanmar name in English**	**Part used**	**Extract weigh (g) **②	**Myanmar traditional use**	**Activity**③	**Literature**④	**Other representative research**
My3	*Mansonia gagei* J.R.Drumm①	Malvaceae	Bustard sandalwood	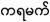	Karamat	woods	3.76	Urein, melena, purgative, skin diseases, hemafecia, paralysis, laxative, fever	Sa*∗∗*	13 (4)	Antifungal, antioxidant and larvicidal, effective compounds includes mansonone C, E, N, and mansorin A, B [18]

My4	*Litsea cubeba *(Lour.) Pers.	Lauraceae	Caraway (Karaway)	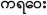	Karaway	fruits	21.00	Digestion, gynecological, regulating menstruation, confectionary flavouring liqueurs	SPI-1*∗∗*	131 (21)	The essential oil of fruits have moderate antibacterial properties [19]

My7	*Luvunga scandens* (Roxb.) Buch.-Ham. ex Wight & Arn.	Rutaceae	Lavang lata	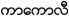	Kakawli	seeds	12.17	Gall-bladder disease, insecticidal, flatulence, phlegmy in throat, hypotension, fever, haematemesis, scorpion poison, insecticidal, anti-itching	SPI-1*∗∗∗*	6 (0)	Essential oil have been reported to be antifungal activity against Keratinophilic fungi [20]

My10	*Mesua ferrea* L.	Calophyllaceae	Ironwood tree	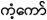	Gangaw	flowers	24.20	Mixed with thana-ka good for skin, insomnia palpitation, dizziness, breathlessness	Pv*∗∗∗*, Sa*∗∗∗*, SPI-1*∗∗*	156 (13)	4-Alkyl- and 4-phenylcoumarins from *Mesua ferrea* as promising multidrug resistant antibacterials [21];

My11	*Myrica nagi* Thunb.①	Pentaphylacaceae	Box myrtle	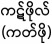	Kat-pho	barks	33.26	Hypertension, coughing, gall-bladder diseases (*∗*)	SPI-1*∗∗∗*	18 (1)	*M. nagi *crude extract posesses antidiarrheal and gut modulatory activities [22]

My21	*Terminalia citrina* Roxb. ex Fleming	Combretaceae	Citrina tree	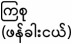	Kyasu (Phan-kha-nge)	fruits	45.18	Asthma, flatulence, burn, toothache	SPI-1*∗∗∗*	8 (1)	Tanins are responsible for antimicrobial activity [23]

My44	*Foeniculum vulgare* Mill.	Apiaceae	Fennel	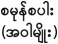	Samon-saba (Awa)	seeds	12.48	Cough, fevers, indigestion, stomachache, apophlegmatisant	SPI-1*∗∗*	933 (128)	Antibacterial property [24]

My45	*Anethum graveolens *L.	Apiaceae	Anise (Sweet fennel)	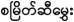	Sameik-si-mwe	seeds	13.05	Spice, medicine to emit unhealthy vapour	SPI-1*∗∗*	410 (60)	Antibacterial property [24]

My49	*Thymus vulgaris *L.	Lamiaceae	Thyme	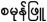	Samon-byu	seeds	13.84	Dysentery, stomach pain, vomiting and diarrhoea used to happen in children (*∗*)	SPI-1*∗∗∗*	1613 (379)	Essential oil of *T. vulgaris *have anitbacterial effect against oral microooganisms in situ [25]

My61	*Myristica fragrans *Houtt.	Myristicaceae	Nutmeg	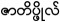	Zadeik-po	seeds	11.39	Tonic, stomachache, piles, nourish blood, arthralgia	SPI-1*∗∗*	380 (33)	3′,4′,7-trihydroxyflavone was major component for treating bactetiial infctiions including mutidrug resistant phenotypes [26]

my67	*Curcuma comosa *Roxb.	Zingiberaceae	Bitter turmeric	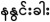	Nawin-kha	roots	17.06	Stomachache, anti-diabetic with honey	Sa*∗∗*	93 (1)	Five diphenylhepranoids were found to be as ematocidal agents [27]

My86	*Garcinia pedunculata *Roxb. ex Buch.-Ham.	Clusiaceae	Boabab	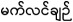	Metlin-chin	fruits	33.20	Constipation and stomachache	SPI-1*∗∗*	29 (1)	The hexane and chloroform extracts of *Garcinia pedunculata *are found to have pronounced inhibitory effect against the tested Gram-positive bacteria [28]

My89	*Centella asiatica *(L.) Urb.	Apiaceae	Asiatic pennywort	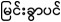	Myin-hkwa-pin	whole plants	12.12	Lungs disease, dysentery, oliguria, hematuria, antidote, influenza, skin disease, hematochezia, wound inflammation (*∗∗*)	SPI-1*∗∗*	960 (35)	Anti-mycobacterial effect against *Mycobacterium tuberculosis* [29]

My90	*Brucea javanica *(L.) Merr.	Simaroubaceae	Java fruit	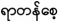	Yar-tan-sae	seeds	8.82	Skin disease, leprosy, scabies, dysentery	SPI-1*∗∗*	224 (1)	A novel antibacterial peptide specific to *Streptococcus pyogenes* was produced from dried fruit protein of *Brucea javanica* [30]

My104	*Cinnamomum bejolghota *(Buch.-Ham.) Sweet	Lauraceae	Cinnamon	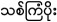	Thit-kyabo	barks	2.28	Digestion, gynecological disease, apoplexy, arthralgia, arthrodynia	SPI-1*∗∗∗*	14 (3)	The essential oil of *Cinnamomum bejolghota* showed promising antibacterial activity [31]

My105	*Coscinium fenestratum *(Goetgh.) Colebr.	Menispermaceae	Tree turmeric	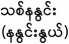	Thit-nan-nwin (Nanwin-nwe)	stems	5.30	Fevers, diabetes, celiac disease, snake bite	SPI-1*∗∗*	65 (2)	Antibacterial activity of *Coscinium fenestratum* is mainly due to the presence of berberine [32]

My108	*Tylophora indica *(Burm. f.) Merr.	Apocynaceae	Country ipecac	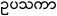	Upa-tha-ka	stems	13.60	Prevent to perspiration, inflammation, asthma	SPI-1*∗∗*	113 (5)	The extracts of *Tylophora indica* acts as a good source of antibiotics against various bacterial pathogens tested and exhibited broad spectrum of antibacterial activity [33]

My109	*Coptis teeta *Wall.	Ranunculaceae	Golden thread	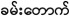	Khandauk	rhizomes	22.50	Mix with *piper nigrum* are used for cough, asthma	Sa*∗∗*	25 (3)	Anti-microbial potential [34]

Note: ① unsolved name based on The Plant List (http://www.theplantlist.org/); ② number of gram extract from 100 g plant material; ③ bioactivities tested in this study (Pv, growth inhibition on *P. vulgaris* CPCC 160013; Sa, growth inhibition on *S. aureus* ATCC 25923; SPI-1, inhibitory activities against the secretion of the *Salmonella* pathogenicity island 1 (SPI-1) effector proteins of *S. enterica* serovar Typhimurium UK-1*χ*8956. SipA/B/C/D, SPI-1 effector proteins). *∗∗* and *∗∗∗* indicate moderate and significant effects, respectively; ④number of literatures retried from Web of Science (http://apps.webofknowledge.com/) of the species studied; numbers in brackets indicate the number of literature related on antimicrobial and/or antibacterial research.

## Data Availability

The data used to support the findings of this study are included in the paper and within the supplementary information file.

## Supplementary Materials

Supplementary Material Fig S1 and Table S1 are provided as the supplementary materials. Fig S1 presents the screening of 93 medicinal Myanmar plants for their effects on the secretion of the* Salmonella* pathogenicity island 1 (SPI-1) effector proteins of* Salmonella enterica* serovar Typhimurium UK-1 *λ*8956. Table S1 provides the brief review of reported chemical constituents of the 18 traditional medicinal plants with antibacterial and anti-T3SS activities.
