# Supplementary material for: Investigation on the Antibacterial and Anti-T3SS Activity of Traditional Myanmar Medicinal Plants
Source: Evid Based Complement Alternat Med. 2018 Oct 9;2018:2812908. doi: 10.1155/2018/2812908 (PMC6198585; doi:10.1155/2018/2812908)
Supplement: Supplementary Material — Fig S1 and Table S1 are provided as the supplementary materials. Fig S1 presents the screening of 93 medicinal Myanmar plants for their effects on the secretion of the Salmonella pathogenicity island 1 (SPI-1) effector proteins of Salmonella enterica serovar Typhimurium UK-1 λ8956. Table S1 provides the brief review of reported chemical constituents of the 18 traditional medicinal plants with antibacterial and anti-T3SS activities. [file 2812908.f1.pdf]

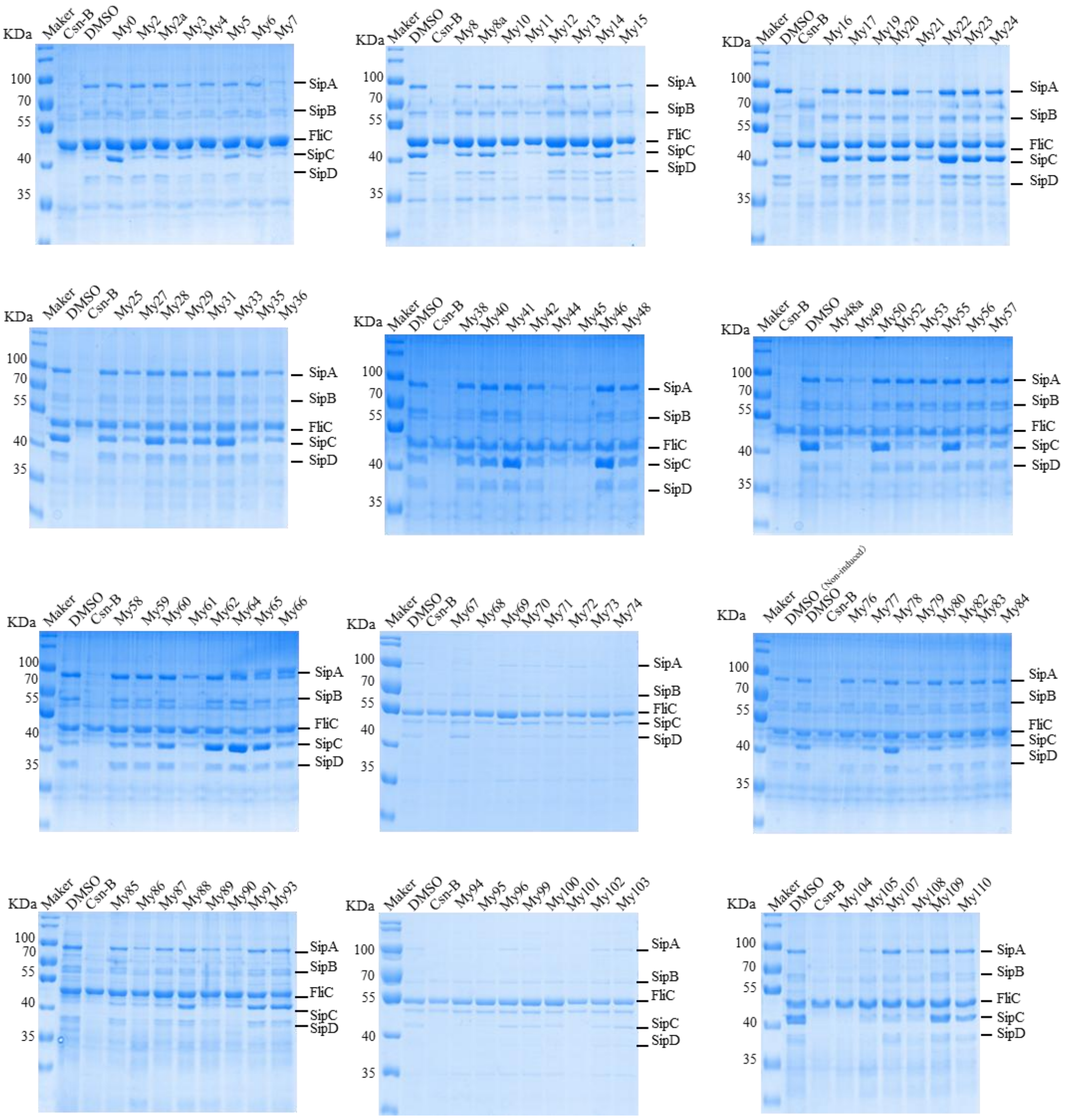

Fig S1. The screening of 93 medicinal Myanmar plant (80 μg/mL) for their effects on the secretion of the Salmonella pathogenicity island 1 (SPI-1) effector proteins of Salmonella enterica serovar Typhimurium UK-1 λ8956. SipA/B/C/D. DMSO, negative control; DMSO (Non-induced), not treated by temperature change; Csn-B, positive control (100 μM).

Table S1. Review of reported chemical constituents of the 18 traditional medicinal plants with antibacterial and anti-T3SS activities

No.	Scientific name	Reported constituents
My3	*Mansonia gagei* J.R.Drumm①	Coumarins: mansonrins A, B,C and Q [1], Ⅰ, Ⅱ, Ⅲ[2]; Sesquiterpenoid naphthoquinone: mansonones C, E, G, H, N, O, P, Q, R and S [1, 3-5]; Neolignan, mansoxetan [5].
My4	*Litsea cubeba* (Lour.) Pers.	1,8-cineole; sabinene; linalool [6]; neral [7]; β-phellandrene; terpinen-4-ol[8]; citral; D-limonene[9]; alkaloids [10, 11].
My7	*Luvunga scandens* (Roxb.) Buch.-Ham. ex Wight & Arn.	Limonoids; Coumarins [12]; Tirucallane triterpenes: 3-oxotirucalla-7,24-dien-21-oic-acid, flindissol [13]; Sobutylglucosinolate; β-sitosterol; stigmasterol [14] fatty-acids [15] xanthyletin [16].
My10	*Mesua ferrea* L.	Pyranoxanthones: mesuaferrin A-C; mesuaxanthones A and B, macluraxanthone, caloxanthone C, 1,5-dihydroxyxanthone, tovopyrifolin C [17-19]; Triterpenoids: β-sitosterol, friedelin, betulinic acid [17]; 4-alkylcoumarins and 4-phenylcoumarins [20, 21].
My11	*Myrica nagi* Thunb.①	Metacyclophane, 13-oxomyricanol [22]; Meta-bridged biphenyls [23]: myricanol, myricanone [24]; Myricitrin, gallic acid, nerolidol, nagilactone C, arjunolic acid, 3-hydroxybenzaldehyde, isovanillin, 4-methoxybenzoic acid, 4-(hydroxymethyl) phenol; β-caryophyllene, β-sitosterol-β-D-glucopyranoside, β-sitosterol, α-caryophyllene, α-cadinol, arjun glucoside, corchoionoside C, α- selinene [25].
My21	*Terminalia citrina* Roxb. ex Fleming	Flavonoids: cyanidin and procyanidin, hydrolyzable ellagitannins; Tannins, gallatannins, and ellagitannins, gallic acid, 12-octadecadienoic acid [26-28]; Santonin, lupeol, cholesterol, campestanol, β-sitosterol, sitostanol [29]; Aalkaloid, Saponins; Cardiac glycosides [30, 31].
My44	*Foeniculum vulgare* Mill.	Phenyl propanoid derivatives; Coumarin derivatives: scopoletin dillapiol, bergapten, imperatorin and psolaren [32] glycosides of stilbene trimer [33]; *trans*-anethole, Lmonene; fenchone; cymene, fatty acids [34-36].
My45	*Anethum graveolens* L.	Carvone, camphor[37], monoterpenes, limonene, flavonoids[38]; Estrogenic [39]; Myristicin[40] [41].
My49	*Thymus vulgaris* L.	*Trans*-4-thuyanol and 4-terpineol [42], 72-methyl-6-methylene-2, 7-octadienol [43]; Thymol [44], carvacro l [45]; Monoterpene: geraniol, alpha-terpineol, 1,8-cineole, borneol, linalool, p-cymene and γ-terpinene [45-48]; Acetophenone glycosides [49]; Saturated hydrocarbons; fatty acids; Aldehydes; Phytosterols; Flavanones [47].
My61	*Myristica fragrans* Houtt.	Neolignans: myticaganal A–C, myrisfrageals A, B; 3′-methoxylicarin B, isodihydrocainatidin, licarin A-E, and dehydrodi-isoeugenol [50-52]; 8-O-4' type neolignan, myrifralignan A-E [53]; 7,7'-epoxylignan nucleus [54]; 7,7'-epoxylignan nucleus, 7',9-epoxilignan [55]; (8*R*,8'*S*)-7-(3,4-methylenedioxyphenyl)-7'-(4-hydroxy-3-methoxyphenyl)-8,8'dimethylbutane [56]; Myristic acid and trimyristin triglyceride, terpenes, Phenylpropanoids, phenolic acids, diarylalkanes, flavonoids, polyphenolics, polycatechins, tannins, anthocyanins [56].
my67	*Curcuma comosa* Roxb.	Diphenylheptanoids: (6*E*)-1,7-diphenylhept-6-en-3-one (DPH1), (4*E*,6*E*)-1,7-diphenylhepta-4,6-dien-3-ol, (6*E*)-1,7-diphenylhept-6-en-3-ol [57, 58]; (3,4-dihydroxyphenyl)-7-(4-hydroxyphenyl)-(6E)-6-hepten-3-ol, 1-(3-hydroxyphenyl)-7-(3,4-dihydroxyphenyl)-3-methoxy-(6E)-6-heptene, (3*R*, 5*R*)-1-(3,4-dihydroxyphenyl)-7-phenyl-heptane-3,5-diol, 7-(3,4 dihydroxyphenyl)-5-hydroxy-1-phenyl-(1*E*)-1-heptene, 1,7-diphenyl-4(E),6(E)-heptadien-3-ol [59-62]; Phloracetophenone glucoside [63]; Comosoxides A-B, comososide [64, 65]; Curcucomosides A-D [66].
My86	*Garcinia pedunculata* Roxb. ex Buch.-Ham.	Benzophenone [67]; xanthone [67-69]; Dulxanthone A, garbogiol, oleanolic acid [68]; Pedunculol, garcinol, cambogin, (-)-hydroxyl citric acid [70].
My89	*Centella asiatica* (L.) Urb.	Isothankunic acid [71]; Triterpenoid, Sterole [72]; Triterpene acid [73]; Flavonoid glycosides [74]; gallic acid, benzophenones, pedenculol, garcinol, and cambogin [75]; 3-isooctadecanyl-4-hydroxy-alpha-pyrone [76]; Triterpene: madecassoside (MS), asiaticoside (AS), madecassic acid (MA) and asiatic acid (AA); asiaticoside, adecassoside [77].
My90	*Brucea javanica* (L.) Merr.	Quassinoid: bruceantin, bruceantinol, bruceins A-G and Q, brucein E, bruceosides A-C, brusatol, dehydrobruceantinol, dehydrobruceins A and B, dehydrobrusatol, dihydrobrucein A, yadanzigan, Bruceoside-A-E, M [78-81]; Dehydrobruceolides [82]; Yadanziosides A-P, N [83, 84]; Alkaloids [85]; Lignan [81]; Terpenoids[81]; Triterpenoid [86].

My104	*Cinnamomum bejolghota* (Buch.-Ham.) Sweet	Carbohydrate, flavanoid glycoside, lignim, tannin, phenolic [87]; *p*-cymene, 1,8-cineole, β-phellendrene, linalool, camphor, terpine-4-ol, a-terpineol, linalyl, acetate, β-caryophyllene [88].
My105	*Coscinium fenestratum* (Goetgh.) Colebr.	Alkaloid: oxypalmatine,(-)-8-oxotetrahydrothalifendine, (-)-8 oxoisocorypalmine [89, 90], berberine [91], berlambine, dihydroberlambine, noroxyhydrastine, steroid [92]; Saponin, Hentriacontane; sitosterol glucoside, palmitic acid, oleic acid [93].
My108	*Tylophora indica* (Burm. f.) Merr.	Alkaloids: tylophorines A-J, 3-O-demethyl tylophorinidine [94-96].
My109	*Coptis teeta* Wall.	Alkaloids: berberine, jateorhizine, palmatine [97], organic acids: quinic, acetic, formic, tartaric, malic, succinic and oxalic acid [98].

Note: ① unsolved name based on The Plant List (http://www.theplantlist.org/);

Reference:

1. P. Tiew, A. Puntumchai, U. Kokpol, and W. Chavasiri. "Coumarins from the heartwoods of Mansonia gagei Drumm.," *Phytochemistry, vol.* 60, no. 8, pp. 773-776.
2. M.A. Baghdadi, F.A. Al-Abbasi, A.M. El-Halawany, A.H. Aseeri, and A.M. Al-Abd. "Anticancer Profiling for Coumarins and Related O-Naphthoquinones from Mansonia gagei against Solid Tumor Cells In Vitro," *Molecules, vol.* 23, no. 5.
3. P. Tiew, J.R. Ioset, U. Kokpol, et al. "Four new sesquiterpenoid derivatives from the heartwood of Mansonia gagei," *Journal Of Natural Products, vol.* 65, no. 9, pp. 1332-1335.
4. A.M. El-Halawany, M.H. Chung, C.M. Ma, K. Komatsua, T. Nishihara, and M. Hattori. "Anti-estrogenic activity of mansorins and mansonones from the heartwood of Mansonia gagei DRUMM.," *Chemical & Pharmaceutical Bulletin, vol.* 55, no. 9, pp. 1332-1337.
5. P. Tiew, H. Takayama, M. Kitajima, N. Aimi, U. Kokpol, and W. Chavasiri. "A novel neolignan, mansoxetane, and two new sesquiterpenes, mansonones R and S, from Mansonia gagei," *Tetrahedron Letters, vol.* 44, no. 35, pp. 6759-6761.
6. L.C. Son, D.N. Dai, T.D. Thang, D.D. Huyen, and I.A. Ogunwande. "Analysis of the Essential Oils from Five Vietnamese Litsea Species (Lauraceae)," *Journal Of Essential Oil Bearing Plants, vol.* 17, no. 5, pp. 960-971.
7. C.L. Ho, O. Jie-Ping, Y.C. Liu, et al. "Compositions and in vitro Anticancer activities of the Leaf and Fruit Oils of Litsea cubeba from Taiwan," *Natural Product Communications, vol.* 5, no. 4, pp. 617-620.
8. H. Wang and Y. Liu. "Chemical composition and antibacterial activity of essential oils from different parts of Litsea cubeba," *Chem Biodivers, vol.* 7, no. 1, pp. 229-235.
9. K. Yang, C.F. Wang, C.X. You, et al. "Bioactivity of essential oil of Litsea cubeba from China and its main compounds against two stored product insects," *Journal Of Asia-Pacific Entomology, vol.* 17, no. 3, pp. 459-466.
10. T. Feng, Y. Xu, X.H. Cai, Z.Z. Du, and X.D. Luo. "Antimicrobially active isoquinoline alkaloids from Litsea cubeba," *Planta Med, vol.* 75, no. 1, pp. 76-79.
11. M. Tomita, S.T. Lu, P.K. Lan, and F.M. Lin. "[Studies on the alkaloids of Formosan lauraceous plants. V. alkaloids of litsea cubeba persoon]," *Yakugaku Zasshi, vol.* 85, no. 7, pp. 593-596.
12. T.P. Nguyen, N.P. Minh, T.B. Dat, et al. "Limonoid from the rhizomes of Luvunga scandens (Roxb.) Buch. Ham," *Nat Prod Res, vol.* 31, no. 19, pp. 2281-2285.
13. P.N.H. Al-Zikri, M. Taher, D. Susanti, et al. "Cytotoxic tirucallane triterpenes from the stem of Luvunga scandens," *Revista Brasileira De Farmacognosia-Brazilian Journal Of Pharmacognosy, vol.* 24, no. 5, pp. 561-564.
14. P. Sirinut, A. Petchkongkeaw, J. Romsaiyud, S. Prateeptongkum, and P. Thongyoo. "Phytochemical

Constituents from the Root of Luvunga Scandens and Biological Activity Evaluation," *Natural Product Communications, vol.* 12, no. 9, pp. 1483-1484.
15. R. Jain and S.C. Garg. "Polarographic Analysis Of Fatty-Acids Obtained From the Fruits Of Luvunga-Scandens Roxb," *National Academy Science Letters-India, vol.* 10, no. 5, pp. 173-175.
16. E. Spath, P.K. Bose, E. Dobrovolny, and A. Mookerjee. "The isolation of xanthyletin from Luvunga scandens Ham," *Berichte Der Deutschen Chemischen Gesellschaft, vol.* 72, pp. 1450-1452.
17. G.C.L. Ee, S.S. Teh, M. Rahmani, Y.H. Taufiq-Yap, R. Go, and S.H. Mah. "A New Furanoxanthone from the Root Bark of Mesua ferrea," *Letters In Organic Chemistry, vol.* 9, no. 6, pp. 457-459.
18. S.S. Teh, G.C.L. Ee, and S.H. Mah. "Chemical Constituents and New Xanthone Derivatives from Mesua ferrea and Mesua congestiflora," *Asian Journal Of Chemistry, vol.* 25, no. 15, pp. 8780-8784.
19. Govindac.Tr, B.R. Pai, Subraman.Ps, U.R. Rao, and Muthukum.N. "Constituents Of Mesua Ferrea L .I. Mesuaxanthone a And Mesuaxanthone B," *Tetrahedron, vol.* 23, no. 1, pp. 243-&.
20. T.R. Govindachari, B.R. Pai, P.S. Subramaniam, U.R. Rao, and N. Muthukumaraswamy. "Constituents Of Mesua Ferrea L .2. Ferruol a a New 4-Alkylcoumarin," *Tetrahedron, vol.* 23, no. 10, pp. 4161-+.
21. L. Verotta, E. Lovaglio, G. Vidari, et al. "4-Alkyl- and 4-phenylcoumarins from Mesua ferrea as promising multidrug resistant antibacterials," *Phytochemistry, vol.* 65, no. 21, pp. 2867-2879.
22. K.E. Malterud and T. Anthonsen. "13-Oxomyricanol, a New [7.0]-Metacyclophane From Myrica-Nagi," *Phytochemistry, vol.* 19, no. 4, pp. 705-707.
23. R.V. Campbell, L. Crombie, B. Tuck, and D.A. Whiting. "Isolation And Structure Of New Meta-Bridged Biphenyls From Myrica-Nagi," *Journal Of the Chemical Society D-Chemical Communications*, no. 18, pp. 1206-&.
24. M.J. Begley, R.V. Campbell, L. Crombie, B. Tuck, and D.A. Whiting. "Constitution And Absolute Configuration Of Meta,Meta-Bridged, Strained Biphenyls From Myrica-Nagi - X-Ray Analysis Of 16-Bromomyricanol," *Journal Of the Chemical Society C-Organic*, no. 21, pp. 3634-&.
25. S.K. Middha, A.K. Goyal, A. Bhardwaj, et al. "In silico exploration of cyclooxygenase inhibitory activity of natural compounds found in Myrica nagi using LC-MS," *Symbiosis, vol.* 70, no. 1-3, pp. 169-178.
26. T.F. Ko, Y.M. Weng, and R.Y.Y. Chiou. "Squalene content and antioxidant activity of Terminalia catappa leaves and seeds," *Journal Of Agricultural And Food Chemistry, vol.* 50, no. 19, pp. 5343-5348.
27. O. Lasekan, K. Alfi, and K.A. Abbas. "Volatile Compounds Of Roasted And Steamed Malaysian Tropical Almond Nut (Terminalia Catappa L.)," *International Journal Of Food Properties, vol.* 15, no. 5, pp. 1120-1132.
28. E.Y.A. Salih, P. Fyhrquist, A.M.A. Abdalla, et al. "LC-MS/MS Tandem Mass Spectrometry for Analysis of Phenolic Compounds and Pentacyclic Triterpenes in Antifungal Extracts of Terminalia brownii (Fresen)," *Antibiotics-Basel, vol.* 6, no. 4.
29. B. Ladele, S. Kpoviessi, H. Ahissou, et al. "Chemical composition and nutritional properties of Terminalia catappa L. oil and kernels from Benin," *Comptes Rendus Chimie, vol.* 19, no. 7, pp. 876-883.
30. M.J. Kaneria, K.D. Rakholiya, L.R. Marsonia, R.A. Dave, and B.A. Golakiya. "Nontargeted metabolomics approach to determine metabolites profile and antioxidant study of Tropical Almond (Terminalia catappa L.) fruit peels using GC-QTOF-MS and LC-QTOF-MS," *J Pharm Biomed Anal, vol.* 160, pp. 415-427.
31. T.D. Baratelli, A.C.C. Gomes, L.A. Wessjohann, R.M. Kuster, and N.K. Simas. "Phytochemical and allelopathic studies of Terminalia catappa L. (Combretaceae)," *Biochemical Systematics And Ecology,*

vol. 41, pp. 119-125.
32. Y.S. Kwon, W.G. Choi, W.J. Kim, et al. "Antimicrobial constituents of Foeniculum vulgare," *Arch Pharm Res, vol.* 25, no. 2, pp. 154-157.
33. M. Ono, Y. Ito, J. Kinjo, S. Yahara, T. Nohara, and Y. Niiho. "4 New Glycosides Of Stilbene Trimer From Foeniculi-Fructus (Fruit Of Foeniculum-Vulgare Miller)," *Chemical & Pharmaceutical Bulletin, vol.* 43, no. 5, pp. 868-871.
34. M.M. Ozcan and J.C. Chalchat. "Comparison Of Chemical Composition Of Essential Oil Obtained From Different Parts Of Foeniculum Vulgare Ssp. Piperitum Used as Condiment," *Journal Of Food Biochemistry, vol.* 34, no. 6, pp. 1268-1274.
35. B. Muckensturm, D. Foechterlen, J.P. Reduron, P. Danton, and M. Hildenbrand. "Phytochemical and chemotaxonomic studies of Foeniculum vulgare," *Biochemical Systematics And Ecology, vol.* 25, no. 4, pp. 353-358.
36. B. Cetin, H. Ozer, A. Cakir, et al. "Antimicrobial Activities of Essential Oil and Hexane Extract of Florence Fennel [Foeniculum vulgare var. azoricum (Mill.) Thell.] Against Foodborne Microorganisms," *Journal Of Medicinal Food, vol.* 13, no. 1, pp. 196-204.
37. M.K.K. Chahal, D. Kataria, and A. Kumar. "Transformation, characterization and bioassay of some compounds from Anethum graveolens L. seed oil: Structure activity relationship," *Allelopathy Journal, vol.* 42, no. 2, pp. 219-230.
38. M. Monsefi and R. Yadollahi. "Effects of Anethum graveolens L. (dill) Seed and Leaf Aqueous Extracts on the Growth of Mammary Gland Alveolar Buds of Rat," *Iranian Journal Of Science And Technology Transaction a-Science, vol.* 41, no. A1, pp. 121-127.
39. G.Q. Zheng, P.M. Kenney, and L.K.T. Lam. "Anethofuran, Carvone, And Limonene - Potential Cancer Chemopreventive Agents From Dill Weed Oil And Caraway Oil," *Planta Med, vol.* 58, no. 4, pp. 338-341.
40. B. Bonnlander and P. Winterhalter. "9-Hydroxypiperitone beta-D-glucopyranoside and other polar constituents from dill (Anethum graveolens L.) herb," *J Agric Food Chem, vol.* 48, no. 10, pp. 4821-4825.
41. M. Stavri and S. Gibbons. "The antimycobacterial constituents of dill (Anethum graveolens)," *Phytother Res, vol.* 19, no. 11, pp. 938-941.
42. R. Granger, J. Passet, and M.C. Pinede. "Trans-4-Thuyanol And 4-Terpineol In Thymus Vulgaris L," *Comptes Rendus Hebdomadaires Des Seances De L Academie Des Sciences Serie D, vol.* 267, no. 22, pp. 1886-&.
43. R. Granger, J. Passet, and J.P. Girard. "72-Methyl-6-Methylene-2, 7-Octadienol Isolated From Thymus-Vulgaris," *Phytochemistry, vol.* 11, no. 7, pp. 2301-&.
44. W. Abdelli, F. Bahri, A. Romane, et al. "Chemical Composition and Anti-inflammatory Activity of Algerian Thymus vulgaris Essential Oil," *Natural Product Communications, vol.* 12, no. 4, pp. 611-614.
45. J.D. Thompson, J.C. Chalchat, A. Michet, Y.B. Linhart, and B. Ehlers. "Qualitative and quantitative variation in monoterpene co-occurrence and composition in the essential oil of Thymus vulgaris chemotypes," *Journal Of Chemical Ecology, vol.* 29, no. 4, pp. 859-880.
46. P. Vernet, P.H. Gouyon, and G. Valdeyron. "Genetic-Control Of the Oil Content In Thymus-Vulgaris L - a Case Of Polymorphism In a Biosynthetic Chain," *Genetica, vol.* 69, no. 3, pp. 227-231.
47. M.D. Guillen and M.J. Manzanos. "Composition of the extract in dichloromethane of the aerial parts of a Spanish wild growing plant Thymus vulgaris L.," *Flavour And Fragrance Journal, vol.* 13, no. 4, pp. 259-262.

48. M. Catauro, F. Bollino, E. Tranquillo, et al. "Chemical analysis and anti-proliferative activity of Campania Thymus Vulgaris essential oil," *Journal Of Essential Oil Research, vol.* 29, no. 6, pp. 461-470.
49. M.F. Wang, H. Kikuzaki, C.C. Lin, et al. "Acetophenone glycosides from thyme (Thymus vulgaris L.)," *Journal Of Agricultural And Food Chemistry, vol.* 47, no. 5, pp. 1911-1914.
50. P. Chumkaew and T. Srisawat. "New neolignans from the seeds of Myristica fragrans and their cytotoxic activities," *J Nat Med*.
51. X.W. Yang, X. Huang, and M. Ahmat. "[New neolignan from seed of Myristica fragrans]," *Zhongguo Zhong Yao Za Zhi, vol.* 33, no. 4, pp. 397-402.
52. G.Y. Cao, X.W. Yang, W. Xu, and F. Li. "New inhibitors of nitric oxide production from the seeds of Myristica fragrans," *Food Chem Toxicol, vol.* 62, pp. 167-171.
53. G.Y. Cao, W. Xu, X.W. Yang, F.J. Gonzalez, and F. Li. "New neolignans from the seeds of Myristica fragrans that inhibit nitric oxide production," *Food Chem, vol.* 173, pp. 231-237.
54. P.H. Nguyen, T.V. Le, H.W. Kang, et al. "AMP-activated protein kinase (AMPK) activators from Myristica fragrans (nutmeg) and their anti-obesity effect," *Bioorg Med Chem Lett, vol.* 20, no. 14, pp. 4128-4131.
55. B.S. Min, T.D. Cuong, T.M. Hung, B.K. Min, B.S. Shin, and M.H. Woo. "Inhibitory Effect of Lignans from Myristica fragrans on LPS-induced NO Production in RAW264.7 Cells," *Bulletin Of the Korean Chemical Society, vol.* 32, no. 11, pp. 4059-4062.
56. E.A. Abourashed and A.T. El-Alfy. "Chemical diversity and pharmacological significance of the secondary metabolites of nutmeg (Myristica fragrans Houtt.)," *Phytochemistry Reviews, vol.* 15, no. 6, pp. 1035-1056.
57. T.M. Jurgens, E.G. Frazier, J.M. Schaeffer, et al. "Novel Nematocidal Agents From Curcuma-Comosa," *Journal Of Natural Products, vol.* 57, no. 2, pp. 230-235.
58. J. Su, K. Sripanidkulchai, A. Suksamrarn, Y. Hu, P. Piyachuturawat, and B. Sripanidkulchai. "Pharmacokinetics and organ distribution of diarylheptanoid phytoestrogens from Curcuma comosa in rats," *J Nat Med, vol.* 66, no. 3, pp. 468-475.
59. A. Sodsai, P. Piyachaturawat, S. Sophasan, A. Suksamrarn, and M. Vongsakul. "Suppression by Curcuma comosa Roxb. of pro-inflammatory cytokine secretion in phorbol-12-myristate-13-acetate stimulated human mononuclear cells," *International Immunopharmacology, vol.* 7, no. 4, pp. 524-531.
60. R. Kaewamatawong, P. Boonchoong, and N. Teerawatanasuk. "Diarylheptanoids from Curcuma comosa," *Phytochemistry Letters, vol.* 2, no. 1, pp. 19-21.
61. T. Jitsanong, K. Khanobdee, P. Piyachaturawat, and K. Wongprasert. "Diarylheptanoid 7-(3,4 dihydroxyphenyl)-5-hydroxy-1-phenyl-(1E)-1-heptene from Curcuma comosa Roxb. protects retinal pigment epithelial cells against oxidative stress-induced cell death," *Toxicology In Vitro, vol.* 25, no. 1, pp. 167-176.
62. T. Matsumoto, S. Nakamura, S. Nakashima, et al. "Diarylheptanoids with inhibitory effects on melanogenesis from the rhizomes of Curcuma comosa in B16 melanoma cells," *Bioorg Med Chem Lett, vol.* 23, no. 18, pp. 5178-5181.
63. A. Suksamrarn, S. Eiamong, P. Piyachaturawat, and L.T. Byrne. "A phloracetophenone glucoside with choleretic activity from Curcuma comosa," *Phytochemistry, vol.* 45, no. 1, pp. 103-105.
64. S. Srichairat, P. Charoenlertkul, S. Lacharoje, and S. Puangchompoo. "Effects of Curcuma comosa on function and pathological changes of the liver and uterus in ovariectomized rats," *Thai Journal Of Veterinary Medicine, vol.* 38, no. 1, pp. 64-64.
65. F.M. Xu, S. Nakamura, Y. Qu, et al. "Structures of New Sesquiterpenes from Curcuma comosa,"

Chemical & Pharmaceutical Bulletin, vol. 56, no. 12, pp. 1710-1716.
66. R. Chokchaisiri, P. Innok, and A. Suksamrarn. "Flavonoid glycosides from the aerial parts of Curcuma comosa," *Phytochemistry Letters, vol.* 5, no. 2, pp. 361-366.
67. A.V.R. Rao, M.R. Sarma, Venkatar.K, and S.S. Yemul. "Benzophenone And Xanthone with Unusual Hydroxylation Patterns From Heartwood Of Garcinia-Pedunculata," *Phytochemistry, vol.* 13, no. 7, pp. 1241-1244.
68. H.T. Vo, N.T.T. Nguyen, G. Maas, U.R. Werz, H.D. Pham, and L.H.D. Nguyen. "Xanthones from the bark of Garcinia pedunculata," *Phytochemistry Letters, vol.* 5, no. 4, pp. 766-769.
69. H.T. Vo, N.T.N. Ngo, T.Q. Bui, H.D. Pham, and L.H.D. Nguyen. "Geranylated tetraoxygenated xanthones from the pericarp of Garcinia pedunculata," *Phytochemistry Letters, vol.* 13, pp. 119-122.
70. R. Mundugaru, S.K. Sivanesan, P. Udaykumar, et al. "Quality Standardization and Nephroprotective Effect of Garcinia pedunculata Roxb. Fruit rind," *Indian Journal Of Pharmaceutical Education And Research, vol.* 51, no. 4, pp. 713-721.
71. T. Dutta and U.P. Basu. "Terpenoids .3. Isolation Of Isothankuniside + Constitution Of Isothankunic Acid From Centella Asiatica Linn (Urb)," *Indian Journal Of Chemistry, vol.* 6, no. 9, pp. 543-&.
72. B. Pasich, Z. Kowalewski, and A. Socha. "Triterpenoid And Sterole Compounds In Plant Material .13. Isolation Of Asiaticoside From Herb Centella Asiatica (L) Urb Using Cationite," *Dissertationes Pharmaceuticae Et Pharmacologicae, vol.* 20, no. 1, pp. 69-+.
73. H. Pinhas. "Structure Of Madasiatic Acid . A New Triterpene Acid From Centella Asiatica L," *Bulletin De La Societe Chimique De France*, no. 10, pp. 3592-&.
74. N. Prum, B. Illel, and J. Raynaud. "The Flavonoid Glycosides From Centella-Asiatica L (Umbelliferae)," *Pharmazie, vol.* 38, no. 6, pp. 423-423.
75. B. Gunther and H. Wagner. "Quantitative determination of triterpenes in extracts and phytopreparations of Centella asiatica (L) Urban," *Phytomedicine, vol.* 3, no. 1, pp. 59-65.
76. R. Srivastava and Y.N. Shukla. "A disubstituted pyrone from Centella asiatica," *Indian Journal Of Chemistry Section B-Organic Chemistry Including Medicinal Chemistry, vol.* 36, no. 10, pp. 963-964.
77. R. Azerad. "Chemical structures, production and enzymatic transformations of sapogenins and saponins from Centella asiatica (L.) Urban," *Fitoterapia, vol.* 114, pp. 168-187.
78. K.H. Lee, Y. Imakura, and H.C. Huang. "Bruceoside-a, a Novel Anti-Leukemic Quassinoid Glycoside From Brucea-Javanica," *Journal Of the Chemical Society-Chemical Communications*, no. 2, pp. 69-70.
79. K.H. Lee, Y. Imakura, Y. Sumida, R.Y. Wu, I.H. Hall, and H.C. Huang. "Anti-Tumor Agents .33. Isolation And Structural Elucidation Of Bruceoside-a And Bruceoside-B, Novel Anti-Leukemic Quassinoid Glycosides, And Brucein-D And Brucein-E From Brucea-Javanica," *Journal Of Organic Chemistry, vol.* 44, no. 13, pp. 2180-2185.
80. N. Fukamiya, M. Okano, M. Miyamoto, K. Tagahara, and K.H. Lee. "Antitumor Agents .127. Bruceoside-C, a New Cytotoxic Quassinoid Glucoside, And Related-Compounds From Brucea-Javanica," *Journal Of Natural Products, vol.* 55, no. 4, pp. 468-475.
81. L. Luyengi, N. Suh, H.H.S. Fong, J.M. Pezzuto, and A.D. Kinghorn. "A lignan and four terpenoids from Brucea javanica that induce differentiation with cultured HL-60 promyelocytic leukemia cells," *Phytochemistry, vol.* 43, no. 2, pp. 409-412.
82. F.A. Darwish, F.J. Evans, and J.D. Phillipson. "Bruceolides And Dehydrobruceolides From Fijian Brucea Javanica," *Planta Med, vol.* 39, no. 3, pp. 232-233.
83. T. Sakaki, S. Yoshimura, T. Tsuyuki, T. Takahashi, T. Honda, and T. Nakanishi. "2 New Quassinoid

Glycosides, Yadanzioside-N And Yadanzioside-O Isolated From Seeds Of Brucea-Javanica (L) Merr," *Tetrahedron Letters, vol.* 27, no. 5, pp. 593-596.
84. T. Sakaki, S. Yoshimura, M. Ishibashi, et al. "New Quassinoid Glycosides, Yadanziosides a-H, From Brucea-Javanica," *Chemical & Pharmaceutical Bulletin, vol.* 32, no. 11, pp. 4702-4705.
85. K.C.S. Liu, S.L. Yang, M.F. Roberts, and J.D. Phillipson. "Canthin-6-One Alkaloids From Cell-Suspension Cultures Of Brucea-Javanica," *Phytochemistry, vol.* 29, no. 1, pp. 141-143.
86. J.H. Liu, J.J. Qin, H.Z. Jin, et al. "A new triterpenoid from Brucea javanica," *Arch Pharm Res, vol.* 32, no. 5, pp. 661-666.
87. B. Gogoi, B.B. Kakoti, N. Sharma, and S. Borah. "Pharmacognostic and preliminary phytochemical evaluation of Cinnamomum bejolghota (Buch.-Ham.) Sweet bark," *Indian Journal Of Natural Products And Resources, vol.* 7, no. 1, pp. 59-64.
88. A. Baruah and S.C. Nath. "Taxonomic status and composition of stem bark oil of a variant of Cinnamomum bejolghota (Lauraceae) from Northeast India," *Nordic Journal Of Botany, vol.* 21, no. 6, pp. 571-576.
89. S. Malhotra, S.C. Taneja, and K.L. Dhar. "Minor Alkaloid From Coscinium-Fenestratum," *Phytochemistry, vol.* 28, no. 7, pp. 1998-1999.
90. P.M.M. Pinho, M.M.M. Pinto, A. Kijjoa, K. Pharadai, J.G. Diaz, and W. Herz. "Protoberberine Alkaloids From Coscinium-Fenestratum," *Phytochemistry, vol.* 31, no. 4, pp. 1403-1407.
91. S. Narasimhan and G.M. Nair. "Cytotoxic effect of Coscinium fenestratum (Gaertn.) Colebr. and its active principle berberine on L929 cells," *Medicinal Chemistry Research, vol.* 14, no. 2, pp. 118-124.
92. P. Deevanhxay, M. Suzuki, N. Maeshibu, H. Li, K. Tanaka, and S. Hirose. "Simultaneous characterization of quaternary alkaloids, 8-oxoprotoberberine alkaloids, and a steroid compound in Coscinium fenestratum by liquid chromatography hybrid ion trap time-of-flight mass spectrometry," *J Pharm Biomed Anal, vol.* 50, no. 3, pp. 413-425.
93. K. Das, R. Dang, G. Sivaraman, and R.P. Ellath. "Phytochemical Screening for Various Secondary Metabolites, Antioxidant, and Anthelmintic Activity of Coscinium fenestratum Fruit Pulp: A New Biosource for Novel Drug Discovery," *Turkish Journal Of Pharmaceutical Sciences, vol.* 15, no. 2, pp. 156-165.
94. K.V. Rao, R.A. Wilson, and B. Cummings. "Alkaloids Of Tylophora .3. New Alkaloids Of Tylophora-Indica (Burm) Merrill And Tylophora-Dalzellii Hook F," *Journal Of Pharmaceutical Sciences, vol.* 60, no. 11, pp. 1725-&.
95. C. Gopalakrishnan, D. Shankaranarayanan, S.K. Nazimudeen, and L. Kameswaran. "Effect Of Tylophorine, a Major Alkaloid Of Tylophora-Indica, on Immunopathological And Inflammatory Reactions," *Indian Journal Of Medical Research, vol.* 71, no. Jun, pp. 940-948.
96. M. Ali, S.H. Ansari, and J.S. Qadry. "Rare Phenanthroindolizidine Alkaloids And a Substituted Phenanthrene, Tyloindane, From Tylophora-Indica," *Journal Of Natural Products, vol.* 54, no. 5, pp. 1271-1278.
97. R. Chatterjee, M.P. Guha, and A. Chatterjee. "Plant Alkaloids .2. Coptis-Teeta, Wall," *Journal Of the Indian Chemical Society, vol.* 29, no. 2, pp. 97-100.
98. D.M. Li, L.L. Zhou, Q.W. Wang, and Y. He. "Determination of organic acids for quality evaluation in Coptis herbs by ion chromatography," *3 Biotech, vol.* 8, no. 6.
